# Performance Comparison of a Laterally-Fed Membrane Chromatography (LFMC) Device with a Commercial Resin Packed Column

**DOI:** 10.3390/membranes9110138

**Published:** 2019-10-29

**Authors:** Pedram Madadkar, Rahul Sadavarte, Raja Ghosh

**Affiliations:** Department of Chemical Engineering, McMaster University, 1280 Main Street W., Hamilton, ON L8S 4L8, Canada; madadkp@mcmaster.ca (P.M.); sadavarh@mcmaster.ca (R.S.)

**Keywords:** membrane chromatography, laterally-fed membrane chromatography, resin column, chromatography, monoclonal antibody, charge variants

## Abstract

The use of conventional membrane adsorbers such as radial flow devices is largely restricted to flow-through applications, such as virus and endotoxin removal, as they fail to give acceptable resolution in bind-and-elute separations. Laterally-fed membrane chromatography or LFMC devices have been specifically developed to combine high-speed with high-resolution. In this study, an LFMC device containing a stack of strong cation exchange membranes was compared with an equivalent resin packed column. Preliminary characterization experiments showed that the LFMC device had a significantly greater number of theoretical plates per metre than the column. These devices were used to separate a ternary model protein mixture consisting of ovalbumin, conalbumin and lysozyme. The resolution obtained with the LFMC device was better than that obtained with the column. For instance, the LFMC device could resolve lysozyme dimer from lysozyme monomer, which was not possible using the column. In addition, the LFMC device could be operated at lower pressure and at significantly higher flow rates. The devices were then compared based on an application case study, i.e., preparative separation of monoclonal antibody charge variants. The LFMC device gave significantly better separation of these variants than the column.

## 1. Introduction

Chromatography remains the standard separation technique for high-resolution separation of biopharmaceuticals, despite some significant shortcomings [1,2,3,4]. The main drawback associated with a resin column is the decrease in resolution with increase in flow rate. This is primarily due to diffusion being the rate-limiting solute transport mechanism. Therefore, columns have to be operated at low flow rates, which reduces productivity [5,6]. Considering that ancillary operations such as cleaning and regeneration are also limited by flow rate, the overall effective productivity in resin-based chromatography is low. The other downside of resin-based chromatography is the high pressure drop across the column, even at relatively low flow rates. Resin columns are scaled up by increasing the column diameter while maintaining the same bed height. Columns with large diameter to bed height ratios suffer from uneven packing and flow maldistribution, which in turn reduce resolution and thereby reduce both product recovery and productivity [7,8].

In the past couple of decades, membranes [9,10,11], monoliths [12,13], and mix-matrices [14] have been proposed as alternatives to conventional resin-based columns. Membrane chromatography, which uses a stack of adsorptive membranes, typically provides an order of magnitude higher productivity [15,16,17] due to the predominance of convective solute mass transport within the adsorptive membrane stack. Its separation performance is also consistently stable over a wide flow rate range. Moreover, the pressure drop across membrane modules is much lower than that across packed columns. These modules could be used in a single-use manner, which eliminates the need for cleaning and revalidation. All of these factors contribute towards lowering the overall production cost [18,19,20]. Despite such advantages, membrane chromatography is used only in very niche applications such as removal of dilute impurities such as viruses, endotoxins and DNA in the flow-through mode [21,22]. Certain design deficiencies with conventional membrane chromatography devices such as the radial flow module result in poor resolution in multi-solute separations, which makes these devices unsuitable for high-resolution, bind-and-elute separations [23].

In recent years, laterally-fed membrane chromatography (or LFMC) modules, which are suitable for high-resolution separations in the bind-and-elute mode, have been introduced [23,24,25]. These modules house within them stacks of rectangular membrane sheets and are provided with lateral flow channels on both sides for feed addition and permeate removal. The design and fabrication of the latest embodiment of this type of modules is described in detail in a recent paper [26]. The novel design features of an LFMC device ensures that the solute flow path lengths within the device are uniform, the pressure drop is uniformly-balanced, and the solute residence time distribution is narrow [27]. These features contribute towards sharp and symmetrical flow-through and eluted peaks, and thereby high-resolution in separation [23,24,25,26,27]. LFMC devices outperform equivalent commercial radial-flow devices in bind-and-elute separations [23]. Their use for challenging high-resolution applications such as the purification of mono-PEGylated proteins [26], the separation of monoclonal antibody aggregates [28], and the separation of charge variants [29] has been reported. This ability to combine high-resolution with high-productivity was the main motivation for the current study, which involves a head-to-head comparison of an LFMC device with an equivalent commercial packed resin column (i.e., having similar volume and ligand chemistry). The separation of three model proteins was first carried out to enable an objective comparison of separation parameters such as resolution, pressure drop and flow rate. The two devices were then compared based on a case study, i.e., the preparative separation of monoclonal antibody (mAb) charge variants. The monoclonal antibody studied was hIgG1-CD4 or Campath-9 [30], which is a humanized IgG1-type antibody against CD4 antigen and which has been used in human clinical trials for the treatment of rheumatoid arthritis and psoriasis. Charge variants of therapeutic antibodies, which are typically formed by chemical modifications such as oxidation and deamidation, could have different biological and pharmacokinetic profiles [31,32,33]. Cation-exchange chromatography is commonly used to separate mAb charge variants [34,35,36,37,38,39,40,41]. The LFMC device used in this study contained a stack of strong cation exchange S (sulfonate) membrane sheets. It was compared head-to-head with a commercial prepacked column containing SP (sulfopropyl) Sepharose high-performance (SP HP) resin [42]. Both devices had similar bed volume (~5 mL) and a similar interacting charged group, i.e., sulfonic acid. Sepharose HP resin particles range in size from 24–44 µm and have been specifically designed for high-resolution applications. The S membrane used in this study had pores ranging in size from 3–5 µm.

## 2. Materials and Methods

### 2.1. Materials

Ovalbumin (A5503), conalbumin (C7786), lysozyme (L6876), citric acid (C0759), sodium citrate dihydrate (S4641), sodium phosphate monobasic (S0751), sodium phosphate dibasic (S0875), and sodium chloride (S7653) were purchased from Sigma-Aldrich (St. Louis, MO, USA). Humanized monoclonal antibody hIgG1-CD4 (Campath-9) was kindly donated by the Therapeutic Antibody Centre, University of Oxford, UK. HiTrap Sepharose SP HP strong cation exchange columns (5 mL bed volume) were purchased from GE healthcare Life Sciences (Piscataway, NJ, USA). The dynamic binding capacity of the Sepharose SP HP strong cation exchange media is 70 mg lysozyme per mL of resin (manufacturers data). Sartobind S cation-exchange membrane sheets (94IEXS42-001) were purchased from Sartorius Stedim Biotech (Gottingen, Germany). The dynamic binding capacity of the Sartobind S cation exchange media is 25 mg lysozyme per mL of membrane (manufacturers data). Lepage epoxy glue was purchased from Henkel (Dusseldorf, Germany). Weld-on 16 glue was purchased from IPS Corporation (Compton, CA, USA). All buffers and the solutions were prepared using water obtained from a SIMPLICITY 185 water purification unit by Millipore (Molsheim, France).

### 2.2. LFMC Device Design and Fabrication

**Figure 1:**
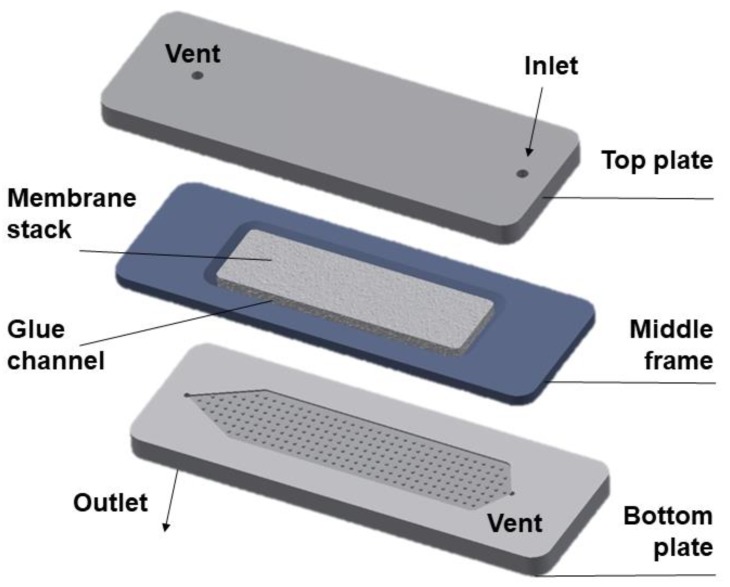
Diagram of a laterally-fed membrane chromatography (LFMC) device.

The design details of the LFMC device (see Figure 1) used in the current study has been reported in an earlier publication [26]. Briefly, the device consisted of two acrylic plates engraved with rectangular channels containing arrays of pillars. Each plate was also provided with two ports, one serving as the inlet/outlet, the other serving as a priming port. The middle frame within which the stack of rectangular membrane sheets was housed (glued in place) was sandwiched between the two plates. The LFMC device used in this study had the bed volume of 4.7 mL. The design specifications are summarized in Table 1. Chromatography experiments using the LFMC device and the column were carried out using an AKTA Prime liquid chromatography system (GE Healthcare Biosciences, QC, Canada).

### 2.3. Theoretical Plates Determination

The number of theoretical plates in the column and the LFMC device were determined using sodium chloride as non-binding tracer. These experiments were carried out with 0.4 M sodium chloride solution as mobile phase and 0.8 M NaCl solution as the salt tracer solution. A 100 µL sample loop was used for sample injection. The number of theoretical plates was calculated based on the attributes of the conductivity peaks as described in the literature [43].

### 2.4. Model Protein Separation 

The ternary model protein system used in this study consisted of ovalbumin (pI 4.5, concentration: 0.2 mg/mL), conalbumin (pI 6.1, concentration: 1.0 mg/mL), and lysozyme (pI 11.0, concentration: 0.5 mg/mL). At the operating condition (i.e., pH 5.5), ovalbumin being negatively charged was obtained in the flow-through, while conalbumin and lysozyme being positively charged were obtained as eluted peaks. The volume of sample injected was 500 µL. Sodium citrate buffer (20 mM, pH 5.5) was used as the binding buffer while the eluting buffer consisted of 0.5 M NaCl solution prepared in the binding buffer. In order to speed up the separations, the linear gradients used for elution were commenced at the same time as the start of sample injection. This was possible due to the ~8 mL lag between initiation of the elution step and the appearance of salt in the eluate for the AKTA Prime liquid chromatography system used in our study. The resolution (R) of the eluted protein peaks was calculated using the following equation:

(1) $$R=1.18\times \frac{V_{R}^{b}-V_{R}^{a}}{W_{0.5}^{a}-W_{0.5}^{b}}$$

In the above equation, *V_R_* denotes retention volume, *w*_0.5_ denotes the peak width at half height, while superscripts *a* and *b* represent the eluted components, i.e., conalbumin and lysozyme, respectively.

### 2.5. Lysozyme Dimer Separation

The lysozyme sample (~0.5 mg/mL concentration) was injected into the LFMC devices using a 2 mL sample loop. The binding buffer was 20 mM sodium phosphate (pH 6.0), while 0.5 M NaCl solution prepared in the binding buffer was used to elute the bound lysozyme. This experiment was carried out at a flow rate of 15 mL/min using a 20 mL linear gradient, which was commenced at the same time as sample injection.

### 2.6. Separation of mAb Charge Variants

The hIgG1-CD4 (pI 8.7) stock solution was diluted by a factor of 10 to a concentration of ~0.5 mg/mL using 20 mM sodium phosphate buffer (pH 6.0). The same buffer was also used as the binding buffer. The eluting buffer consisted of 0.5 M NaCl solution prepared in binding buffer. The volume of sample injected was 2 mL. Shallow linear salt gradients were then used to fractionate the charge variants.

## 3. Results and Discussion

Table 2 compares the efficiency of the LFMC device with the HiTrap Sepharose SP HP column in terms of the calculated theoretical plates per unit bed height at three different flow rates, i.e., 5, 10 and 15 mL/min. The bed volume of the column and the LFMC device being similar, i.e., 5 and 4.7 mL, respectively, the residence times in these devices were similar at the same flow rate. Theoretical plate measurements could not be made at 30 mL/min flow rate using the column and so only data obtained using the LFMC device is shown for this flow rate. Consistent with expectation [7,8], the efficiency of the HiTrap Sepharose SP HP column decreased with the increase in flow rate. However, with the LFMC device, the efficiency increased when the flow rate was increased from 5 to 10 mL/min, and then remained high at 15 mL/min before decreasing at 30 mL/min. This clearly indicated that the LFMC device had a greater operating flow rate range than the column. The higher efficiency of the LFMC device compared to the column was probably due to a combination of the predominantly convective solute transport within the LFMC devices as well as its superior flow distribution attributes, which resulted in a lower degree of dispersion than that within the column. In addition, the smaller size of the membrane pores (i.e., 3–5 µm) compared to the diameter of the Sepharose HP resin particles (i.e., 24–44 µm) could be an important factor. Whether the membrane bed would be more susceptible to blockage on account of the lower effective pore size than the resin bed will be examined in future studies.

**Figure 2:**
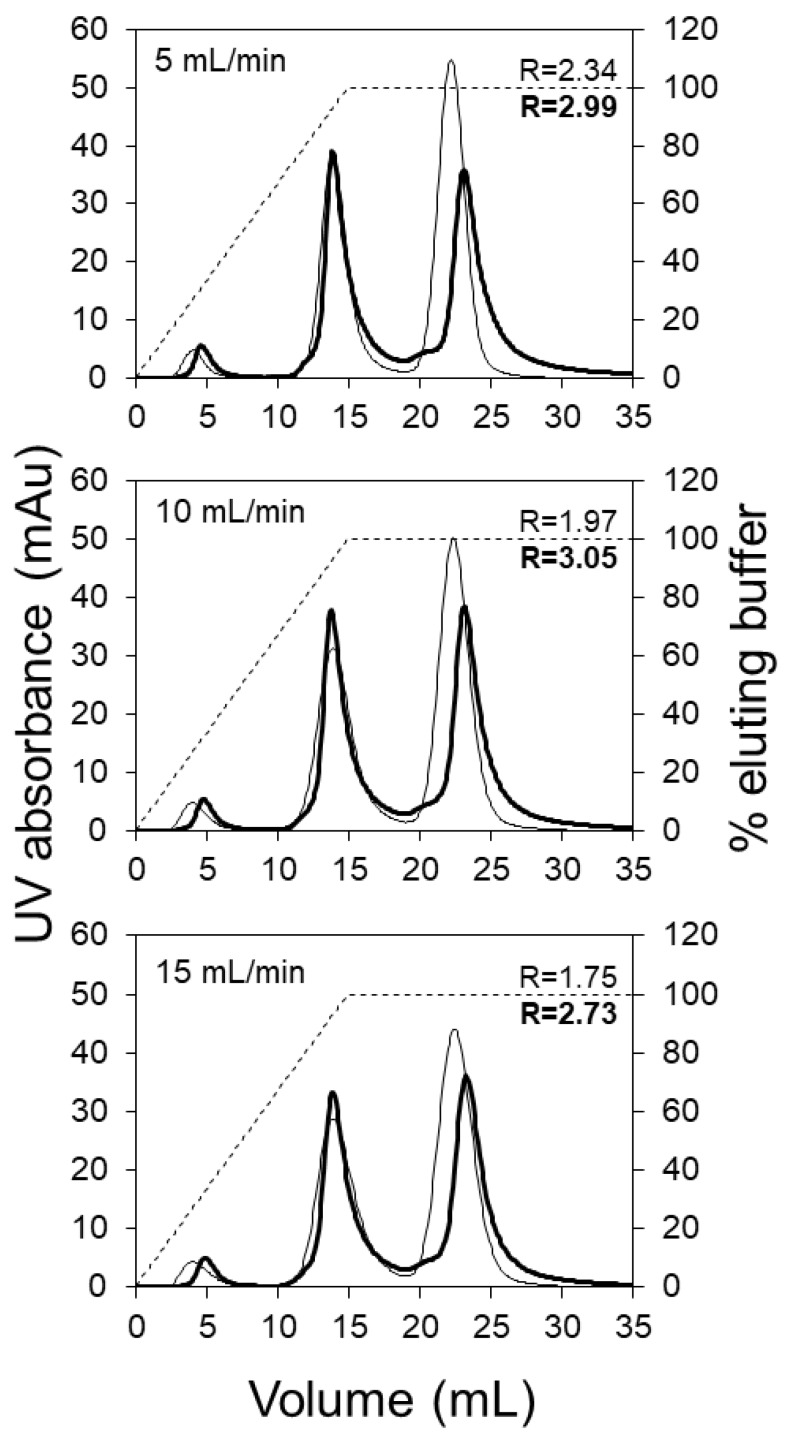
Model protein separation using the LFMC device and HiTrap SP HP column (membrane: Sartorius S cation exchange; membrane bed volume: 4.7 mL; column volume: 5 mL; feed: 0.2 mg/mL ovalbumin, 1.0 mg/mL conalbumin, 0.5 mg/mL lysozyme; sample volume: 500 µL; binding buffer: 20 mM sodium citrate pH 5.5; eluting buffer: binding buffer + 0.5 M NaCl; linear gradient volume: 15 mL; thin line: column; thick line: LFMC device; solid line: UV absorbance; dashed line: elution gradient).

Based on the pI values of the three model proteins and the operating pH selected, ovalbumin should be obtained in the flow through (i.e., around 5 mL effluent volume), followed by the elution of conalbumin and lysozyme, respectively. The chromatograms shown in Figure 2 compare the performance of the HiTrap SP HP (5 mL) column with the LFMC device (4.7 mL) using a 15 mL linear salt gradient, at three different flow rates, i.e., 5, 10 and 15 mL/min. The LFMC device gave very sharp ovalbumin flow through peaks at all the flow rates examined, while the corresponding peaks obtained with the HiTrap column were broader with significant tailing. This difference in the shape and height of the flow-through peaks provide preliminary evidence on the superior hydraulic attributes of the LFMC device. With columns, the radially outward distribution of the liquid in the top header followed by the radially inward collection in the bottom header results in some non-uniformity in the flow path lengths, resulting in broadening of residence time distribution, eventually resulting in peak broadening [8]. While this type of dispersion is usually expected with large-scale columns [8], it was surprising to observe the same happen at the scale examined in this study, i.e., 5 mL bed volume. The conalbumin peaks obtained with both devices were fairly similar. However, the lysozyme peaks were quite different. The retention volume of the lysozyme peak obtained with the LFMC device was consistently greater than that obtained with the column (i.e., conalbumin and lysozyme were better resolved). The conalbumin/lysozyme resolution (*R*) data is also shown in Figure 2. The lysozyme peak height in the chromatograms obtained with the LFMC device was lower and was preceded by a pre-peak, which was not observed in the chromatograms obtained with the resin column. Our initial hypothesis was that the pre-peak indicated the presence of lysozyme dimer, which is typically formed by oxidation [44,45]. Earlier studies have shown that the dimer appears just before the monomer during cation exchange chromatography of a mixture containing the two [46,47].

**Figure 3:**
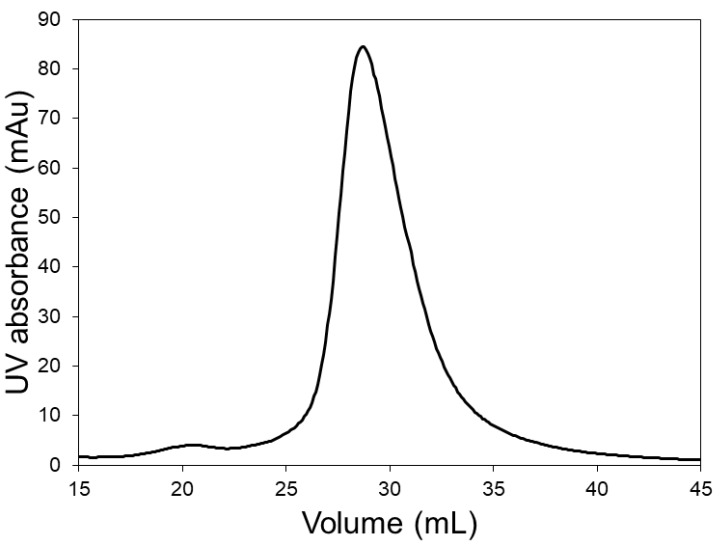
Chromatogram of dimer containing lysozyme sample obtained with the LFMC device (membrane: Sartorius S; membrane bed volume: 4.7 mL; feed: 0.5 mg/mL lysozyme; sample volume: 2 mL; binding buffer: 20 mM sodium phosphate pH 6.0; eluting buffer: binding buffer + 0.5 M NaCl; linear gradient volume: 20 mL; flow rate: 15 mL/min).

In order to investigate whether the pre-peak was indeed due to the presence of lysozyme dimer, chromatography experiments were carried out with the LFMC device using a lysozyme sample. The chromatogram obtained with lysozyme (see Figure 3) clearly shows the lysozyme dimer peak, corresponding to a retention volume of about 21 mL. This residence volume perfectly matched those of the pre-peaks observed in Figure 2. The data shown in Figure 2 and Figure 3 clearly demonstrate the superior resolution capabilities of the LFMC device. While this device could distinguish and resolve the lysozyme dimer from the lysozyme monomer peak, this was clearly not possible using the resin column. The higher lysozyme peaks obtained with the resin column could therefore be attributed to its inability to resolve lysozyme dimer from lysozyme monomer.

The resolution of the eluted proteins obtained with the LFMC device were superior than those obtained with the HiTrap column even though the bed height of the column was almost eight times greater, i.e., 25 mm as opposed to 3.3 mm for the columns. This was consistent with the expectations based on the theoretical plates data obtained with these devices (see Table 2). Table 3 summarizes the pressure drop in the two devices at different flow rates. At the same flow rate, the pressure drop was significantly lower with the LFMC device, clearly indicating its suitability for high-speed separations at high flow rates.

**Figure 4:**
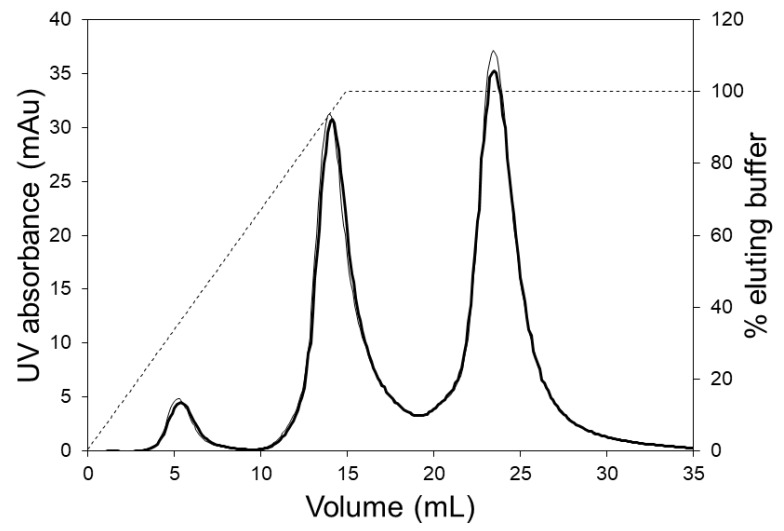
Model protein separation using the LFMC device at high flow rates (membrane: Sartorius S cation exchange; membrane bed volume: 4.7 mL; feed: 0.2 mg/mL ovalbumin, 1.0 mg/mL conalbumin, 0.5 mg/mL lysozyme; sample volume: 500 µL; binding buffer: 20 mM sodium citrate pH 5.5; eluting buffer: binding buffer + 0.5 M NaCl; linear gradient volume: 15 mL; thin line: 25 mL/min flow rate; thick line: 30 mL/min flow rate; solid line: UV absorbance; dashed line: elution gradient).

Figure 4 shows the chromatograms obtained with the LFMC devices at higher flow rates, i.e., 25 and 30 mL/min. These results clearly showed that the LFMC device performed well even at twice the recommended flow rate for the HiTrap column (i.e., 15 mL/min). This attribute is extremely advantageous from a manufacturing point of view as greater productivity could be achieved without significantly compromising on resolution. Interestingly, the lysozyme dimer was not resolved from the lysozyme monomer at these higher flow rates. This was consistent with the expectations based on the decrease in the number of theoretical plates with the LFMC device when the flow rate was increased from 15 to 30 mL/min (see Table 2), i.e., the resolution decreased slightly at the higher flow rates. However, the three principal model proteins, i.e., ovalbumin, conalbumin and lysozyme, were satisfactorily resolved. Such high-resolution separation capabilities at high flow rates have also been reported for monoliths [12,13].

**Figure 5:**
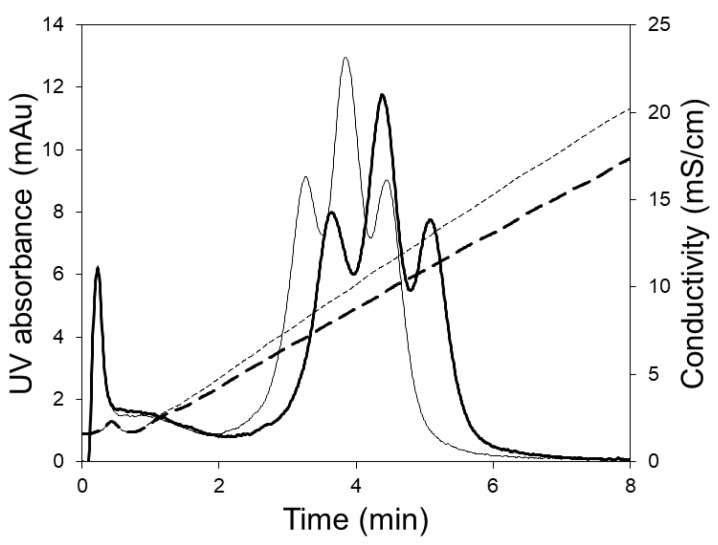
HIgG1-CD4 charge variant separation carried out with the LFMC device using 250 mL (thin line) and 300 mL (thick line) linear salt gradients (membrane: Sartorius S cation exchange; membrane bed volume: 4.7 mL; feed: 0.5 mg/mL HIgG1-CD4; sample volume: 2 mL; binding buffer: 20 mM sodium phosphate pH 6.0; eluting buffer: binding buffer + 0.5 M NaCl; flow rate: 15 mL/min; solid line: UV absorbance; dashed line: conductivity).

Figure 5 shows the chromatograms obtained during the separation of hIgG1-CD4 charge variants using the LFMC device. These experiments were carried out using two different linear gradients, i.e., 250 and 350 mL. The three peaks obtained in each case (from left to right) were respectively the acidic variants peak, the main (or neutral) fraction peak, and the basic variants peak. The same monoclonal antibody (i.e., hIgG1-CD4) sample had been previously studied for the development of an analytical separation technique based on cation exchange membranes, and the presence of the acidic, neutral and basic variants in this had been clearly demonstrated [29]. Shallow gradients (i.e., 250 and 350 mL) were required to resolve the charge gradients since the physicochemical differences between them was very subtle [29]. At a flow rate of 15 mL/min, the separation of the variants could be achieved in 7 min. The conductivity gradient was very stable even when it was very shallow (i.e., 350 mL) and this contributed to the good quality of separation.

**Figure 6:**
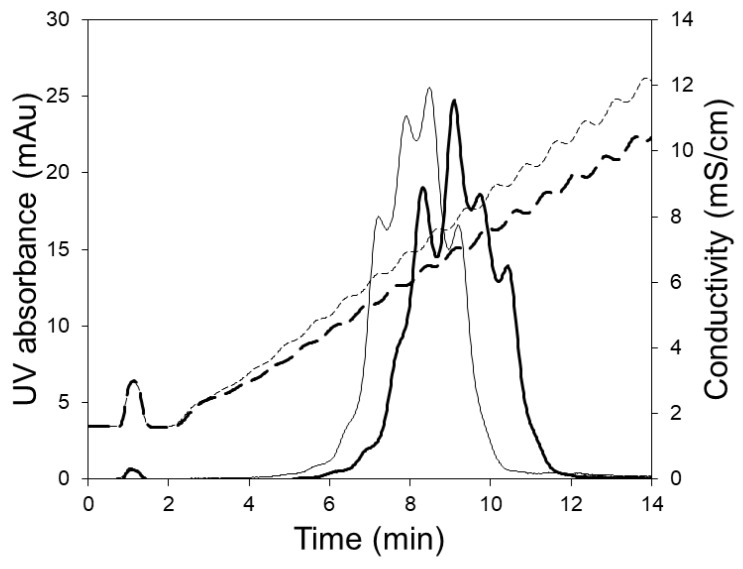
HIgG1-CD4 charge variant separation with HiTrap SP HP column using 250 mL (thin line) and 300 mL (thick line) linear salt gradients (column volume: 5.0 mL; feed: 0.5 mg/mL hIgG1-CD4; sample volume: 2 mL; binding buffer: 20 mM sodium phosphate pH 6.0; eluting buffer: binding buffer + 0.5 M NaCl; flow rate: 15 mL/min, solid line: UV absorbance; dashed line: conductivity).

Figure 6 shows the chromatograms obtained when the 5 mL HiTrap column was used for separating the charge variants. These experiments were carried out at the manufacturers recommended flow rate (5 mL/min) using 250 and 350 mL linear salt gradients. Clearly, the separation of variants obtained using the column was inferior to that obtained with the LFMC device (see Figure 5). The poor separation obtained with the column could be attributed to greater non-uniformity in flow, bigger particle size (relative to the membrane pore size), and the unstable conductivity profiles obtained at the shallow gradients required for this challenging separation. The jagged conductivity profiles (see Figure 6), which were presumably caused by non-uniform flow distribution within the column, contributed significantly towards the jagged and unresolved protein peaks. Thus, while the LFMC device could be operated using very shallow gradients, the same could not be done using the column.

## 4. Conclusions

Our earlier studies had demonstrated the superiority of the LFMC device over stacked-disc and radial flow membrane chromatography devices. The results discussed in this paper clearly demonstrate that superior resolution could be obtained with the LFMC device compared to an equivalent resin based column during separation of a ternary model protein mixture in the bind-and-elute mode. The LFMC device could even resolve lysozyme dimer from lysozyme monomer, which was not possible using the equivalent column. When these devices were used for fractionating monoclonal antibody charge variants using very shallow gradients, the resolution obtained with the LFMC device was significantly superior. Better separation obtained using the LFMC device correlated well with its significantly higher number of theoretical plates in unit length. Superior separation was obtained with the LFMC device despite the fact that it had a significantly lower bed height compared to the column, i.e., 3 mm as opposed to 25 mm. The low bed height of the LFMC device also made it possible to carry out these separations at significantly lower pressure drops. Overall, the LFMC device combined high-resolution in separation of a chromatographic separation, with the high-speed and low pressure attributes of membrane separation. Therefore, it shows significant promise for use in large-scale, high-resolution biopharmaceutical purification and similar applications.

## Figures and Tables

**Table 1:** Design details of the LFMC device.

Membrane Bed Volume (mL)	Number of Membrane Layers	Bed Height (mm)	Membrane Dimensions (mm × mm)	Pillar Array	Outer Dimension of Plate (mm × mm)
4.7	12	3.3	70 × 20	28 × 7	150 × 40

**Table 2:** Number of theoretical plates per metre (N/m) in the HiTrap SP HP column and LFMC device at different flow rates.

Device	Bed Volume (mL)	Flow Rate (mL/min)	Number of Theoretical Plates Per Metre (m^−1^)
HiTrap SP HP	5	5.0	6500
		10.0	4100
		15.0	3600
LFMC	4.7	5.0	21,400
		10.0	25,100
		15.0	24,400
		30.0	18,100

**Table 3:** Pressure drop in HiTrap SP HP column and LFMC device during the separation of model protein mixture at different flow rates.

Device	Bed Volume (mL)	Flow Rate (mL/min)	Pressure Drop (MPa)
HiTrap SP HP	5	5	0.068
		10	0.146
		15	0.232
LFMC	4.7	5	0.037
		10	0.083
		15	0.134
